# Retraction of: Sulfated polysaccharide of *Sepiella maindroni* ink targets Akt and overcomes resistance to the FGFR inhibitor AZD4547 in bladder cancer

**DOI:** 10.18632/aging.206104

**Published:** 2024-09-15

**Authors:** Liping Shan, Wei Liu, Yunhong Zhan

**Affiliations:** 1 Department of Urology, Shengjing Hospital, China Medical University, Shenyang, Liaoning 110004, China; 2 Emergency Department, First Hospital of China Medical University, Shenyang, Liaoning, China

**Keywords:** AZD4547, sulfated polysaccharide of *Sepiella maindroni* ink, bladder cancer, FGFR therapy resistance, xenograft

**This article has been retracted:** Aging has completed its investigation of this paper. Multiple concerns were raised about this paper, including internal duplication and overlap with unrelated papers from different institutions. The internal duplication was found in Transwell migration assay images in **Figure 5D**. The external duplications/overlaps were with articles submitted to journals between April 2018 and June 2019, before the submission date of the article of interest. These include Transwell assay images in **Figure 5D**, where panels 1, 2 and 4 overlap with some of the images in Figure 5B in [1]. Panel 1 also overlaps with images in Figure 1H in [2] and Figure 3F in [3], and panel 5 overlaps with the image in Figure 4A in [4]. In addition, the Western blot Akt panel in **Figure 2D** overlaps with the CDK4 panel in Figure 3D in [5].

The Scientific Integrity office at Aging contacted the authors, but the authors did not respond to requests to clarify, and the paper was retracted based on an Editorial decision. The Scientific Integrity office also notified the authors’ Institutions about this retraction and added their names to an Editorial Warning list.
